# Dormant Cells of *Staphylococcus aureus* Are Resuscitated by Spent Culture Supernatant

**DOI:** 10.1371/journal.pone.0085998

**Published:** 2014-02-11

**Authors:** Ben Pascoe, Lucy Dams, Tom S. Wilkinson, Llinos G. Harris, Owen Bodger, Dietrich Mack, Angharad P. Davies

**Affiliations:** 1 Institute of Life Science, Swansea University College of Medicine, Swansea, United Kingdom; 2 Public Health Wales Microbiology Swansea, Swansea, United Kingdom; 3 Bioscientia Labor Ingelheim, Institut für Medizinische Diagnostik GmbH, Mainz, Germany; University of Iowa Carver College of Medicine, United States of America

## Abstract

We describe the first *in vitro* model of dormancy in *Staphylococcus aureus*, showing that cells are generated which can be resuscitated by addition of spent medium supernatant taken from cultures of the same organism. Over 30 days, culturable counts in dormant cultures of *S. aureus* SH1000 fell from 10^6^–10^7^ cfu/ml to <10 cfu/ml as measured by the Most Probable Number method in liquid culture, while total counts as determined by microscopy, and supported by data from RT-qPCR, remained around 10^6^–10^7^ cells/ml. Supplementing cultures with 25–50% spent medium resulted in a >600-fold increase in bacterial growth. Resuscitation was a specific effect, greatly reduced by boiling or addition of trypsin to the spent supernatant. Supernatant also effected a reduction in lag phase of dormant cultures. SEM demonstrated the presence of small coccoid cells in dormant cultures. The results are similar to those seen with resuscitation promoting factors (Rpfs) in actinobacteria. This is the first time resuscitation has been demonstrated in *Staphylococcus aureus*, which is an important human pathogen. A better understanding of control and reactivation of dormant cells could lead to major improvements in managing staphylococcal infections; resuscitation could be an important step in restoring susceptibility to antibiotic treatment.

## Introduction

Staphylococci form part of the natural human and animal microbial flora. Amongst many virulence attributes they have the ability to form biofilm and to cause chronic infection despite apparently appropriate antibiotic treatment, a clinical phenomenon known as persistence [1]. Tolerance of persister organisms to antibiotics is well recognized and offers resistance to killing, though at the cost of non-proliferation [2]. Eradicating such infections is notoriously difficult in bone, prosthetic device infections, tuberculosis, endocarditis, and chronic lung infections. Prosthetic device infections in particular are most commonly caused by staphylococci, either coagulase-positive (*S. aureus*) or coagulase-negative, since their particular propensity to form biofilm allows them to colonise biomaterials, and persisters have been shown to be present in this context [3], [4]. Antibiotic resistance in biofilms is poorly understood. Planktonic cells originating in biofilm are often susceptible to antibiotics, as are, apparently, many within the biofilm [5]. The persister state may also contribute to immune evasion of biofilm [6]. Survival of the organisms can then cause relapsing infection.

An alteration in metabolism is likely to account at least in part for the decreased antimicrobial susceptibility, and some of these cells may exhibit the characteristics of true dormancy. The term ‘dormancy’ has been used to describe a wide range of different states. Given that dormancy necessarily requires the concept of exit from dormancy, or resuscitation, the definition used for the purposes of this paper is that of Kell and Young: ‘In a state of low metabolic activity and unable to divide or form a colony on an agar plate without a preceding resuscitation phase’ [7]. Although believed to be crucially important clinically, little is known about what triggers dormancy, the metabolic status of dormant cells, or how organisms revert from this state. Such cells are formed at greatest frequency during the stationary phase of growth but even then the majority (about 99%) are non-dormant, suggesting that the dormant state is a kind of altruistic state benefitting the population, rather than the individual [2].

In the actinobacteria, including *M. tuberculosis*, small proteins known as resuscitation-promoting factors (Rpfs) can resuscitate dormant organisms so that they become culturable once more [8], [9], [10], [11], a finding first demonstrated in the organism *Micrococcus luteus*. Active in the picomolar range, Rpfs increase the number of culturable cells from dormant populations at least 100-fold [8]. Rpfs are secreted by the organism and are present in supernatants of exponentially growing cultures, which therefore also exert a resuscitation effect [8]. As well as allowing dormant bacterial cells to grow which cannot grow in their absence, they also decrease the lag phase in cultures to which they are added^8^. Data from bacterial genome sequencing projects have revealed the existence of over 40 examples of *rpf-*like genes in the actinobacteria, including streptomycetes, corynebacteria and mycobacteria [7], [8], [12]. Most organisms contain several representatives. They have been shown to be expressed during human infection [13], and inhibitors of Rpf might have applications as therapeutic agents targeting reactivation of latent tuberculosis [14]. Recently, Lmo186 and Lmo2522 were shown to have Rpf-like activity in the firmicute *Listeria monocytogenes* [15].

In this study, we show that dormant (non-culturable without a preceding resuscitation phase) cells of *S. aureus* SH1000 [16] are produced in a cold starvation model and that spent culture supernatant can resuscitate these cells, and reduce the lag phase, with findings very similar to those in actinobacteria.

## Materials and Methods

### Bacterial Strains

Most experiments were performed with the *S. aureus* SH1000 wild-type strain [16]. Seven other strains of *S. aureus* were also tested. These were *S. aureus* strains 8325-4 (from which SH1000 was derived; sequence type (ST) 8, clonal complex (CC) 8), SA113 (also closely related to SH1000; ST8, CC8) [17], USA300 (a community MRSA strain; ST8, CC8) [18], MW2 (a community MRSA strain; ST1, CC1) [19], MRSA252 (a hospital-associated MRSA strain; ST36, CC30) [20], RN4220 (a laboratory strain; ST8, CC8) [21] and Col (an early clinical MRSA strain; ST250, CC8) [22].

### Media

Tryptone soy broth (TSB) (BD biosciences) medium was used to culture staphylococci for inoculation into dormant cultures. For producing active supernatant, and for culturing staphylococci in the Bioscreen analyser for measurement of lag phase, chemically defined minimal medium composed of glucose, 18 amino acids, two purines and six vitamins was used as described by Hussein *et al* [23]. The rationale for the use of minimal medium for measuring resuscitation and decreases in lag phase was that cells would grow more slowly overall, enabling any differences in growth and/or lag phase to be more easily detected.

### In vitro Model for Dormancy in Staphylococci

Cultures of the *S. aureus* SH1000 wild type strain were used to inoculate TSB medium overnight, diluted 1 in 1000 and grown to an optical density of OD_600_ = ∼0.6. These cells were washed with water by centrifugation before using ∼5–10×10^6^ cells to inoculate 50 ml of sterile distilled water and held at 4°C for up to a month, similar to the method of Wai *et al* [24]. Culturable cell counts were measured periodically on solid agar and also in liquid culture by using 5-tube most probable number (MPN) tests in liquid medium [25]. An aliquot was taken from the bacterial suspension and used to inoculate 5 replicates of 10-fold serial dilutions in TSB medium. The number of turbid tubes observed after incubation at three successive dilutions can be used to read MPN of culturable cells contained within the original suspension from published statistical tables [25]. Total cell counts were obtained by staining the cells with safranin and counting using a haemocytometer under the microscope. The numbers of colony forming units (cfu) were measured over time in this system using both liquid (MPN tests) and solid agar. To support the total count measurement, total *S. aureus* DNA content was also measured in the cultures, by quantitative real-time PCR. To confirm the validity of this approach, and show that DNA content was proportional to cell number, DNA content in spun-down culture supernatant was also measured (to exclude the presence of free DNA), and total DNA content of standard cultures measured and correlated with cfu/ml.

### Resuscitation with spent medium in MPN tests

Spent medium was harvested from the SH1000 wild type *S. aureus* strain grown in minimal medium at intervals until late log phase, the point at which supernatants of actinobacteria likely display the greatest resuscitatory activity and contain the greatest amount of Rpf [26]. Cells were removed by centrifugation and then the supernatant was sterile-filtered with an Ø 0.22 µM filter unit. Aliquots of 100 µl of bacterial cultures from the dormant model at different time points (between 1 and 72 days) were used to inoculate 900 µl fresh minimal medium supplemented with spent medium supernatant. The ratio of spent medium supernatant to fresh minimal medium varied: 100 µl:800 µl for experiments using 10% spent medium supernatant, 250 µl: 650 µl for experiments using 25% spent medium supernatant and 500 µl: 400 µl for experiments using 50% spent medium supernatant. The concentration of cells in the dormant models prior to inoculating the fresh minimal medium was was approximately 10^8^ cfu/ml, giving a final bacterial concentration of approximately 10^7^ cfu/ml. These cultures were then serially diluted in tenfold dilutions to perform 5-tube MPN tests. Negative controls were prepared as above, with 0.9 ml fresh minimal medium and without spent medium supernatant. In order to exclude carry-over of cells in the supernatant (from inadequate filtering), spent medium supernatant and fresh minimal medium were also incubated without inoculating with dormant cells. All cultures were incubated at 37°C for 24 hours and all comparisons were done in at least 3 independent experiments.

### Measurement of lag phase with and without spent medium

The Bioscreen C optical growth analyzer (Lab systems, Finland) was used to monitor growth rates of *S. aureus* strains in wells of a microtitre plate. Cultures had a final volume of 200 µl, consisting of 99 µl fresh minimal medium supplemented with 99 µl spent medium supernatant (prepared as above), inoculated with 2 µl of dormant culture. The final cell concentration in each well was therefore approximately 10^6^ cfu/ml. Negative controls consisted of 198 µl of fresh minimal medium with 2 µl of dormant cultures; negative controls of supplemented and unsupplemented fresh medium without inoculum were also prepared. Experiments were performed on dormant cultures aged 7, 10, 14 and 21 days. Cultures were incubated at 37°C for 2 days (48 hours), eventually forming turbid cultures, and an optical density measurement (OD_600_) was taken every 30 minutes. Each run contained 5 technical replicas. The point at which growth exceeded OD_600_ = 0.1 was noted and the relationship of difference in growth between cultures resuscitated in fresh medium only and supplemented media were analysed for statistical significance. If the differences were binomially distributed then the addition of spent medium had no significant effect. If the supplemented resuscitation media returned a shorter lag phase on enough occasions, giving rise to a P-value less than 0.05 (95% confidence) the difference was deemed to be statistically significant. Identical experiments were carried out using spent medium from the other seven strains of *S. aureus*, and dormant cells generated from the seven other strains.

### Boiling or trypsin digestion of supernatant

In order to demonstrate that the apparent resuscitation was a specific effect, and not simply due to the presence of nutrients, the supernatant was boiled to denature proteins, prior to use in resuscitation experiments. The spent medium supernatant was also subjected to trypsin digestion to denature protein activity: filtered spent medium was digested with 50 µg/ml trypsin from bovine pancreas for 30 minutes at 37°C. Digestion was stopped by addition of 100 µg/ml type I-S trypsin inhibitor from soybean. Resuscitation experiments were as described above, using either 25% boiled or 25% trypsin digested supernatant. Controls were performed as above and used 25% unboiled/undigested supernatant. All comparisons were done in at least 3 independent experiments.

### Ultrafiltration of supernatant

Spent media supernatants were fractionated by ultrafiltration in order to further characterize the active component. Sterile-filtered spent medium was systematically ultrafiltrated through filters of pore sizes 30>10>3 KDa (Millipore UK) and each fraction brought back to the original volume with fresh minimal medium.

### Scanning Electron Microscopy

Samples of dormant cultures and an overnight control culture were prepared for viewing using the Hitachi S4800 High resolution SEM by air drying onto a thin shard of broken glass cover slip. After drying the shards were rinsed three times with 0.1 M PIPES, pH 7.4 for 2 minutes. They were then fixed with 2.5% gluteraldehyde in 0.1 M PIPES, pH 7.4 for 5 minutes, followed by rinsing again with 0.1 M PIPES, pH 7.4 for 2 minutes (3 times). Samples were post-fixed in the dark with 1% osmium tetroxide in 0.1 M PIPES, pH 6.8 for 60 minutes and finally rinsed with double distilled water for 2 minutes (3 times). They were then dehydrated by exposure to increasing amounts of ethanol (up to 100%) for 5 minutes each, followed by 1∶1 ethanol and hexamethyldisilazane (HDMS) for 5 minutes and finally 100% HDMS for 5 min [27]. Samples were air dried before mounting samples onto an aluminum stub for visualization under the microscope.

### Quantitative real-time PCR

Quantitative real-time PCR was used to estimate total genomic DNA within the dormant samples using primers for *nucA* [28], *sa442* [29] (both specific to *S. aureus*) and 16S rRNA (ubiquitous among bacterial species) genes. Primer sequences are shown in Table S1. Primer pairs were tested using 10-fold serial dilutions of genomic DNA to generate a standard curve and determine their amplification efficiency. The amplification efficiency was determined from the slope of each standard curve and only primers that gave an amplification efficiency between 90–105% were used. All real-time PCR reactions were performed using Bioline sensimix (No Rox) kit, reactions were prepared in 25 µl reactions and amplification analysed by Corbett RotorGene-6000. Calibration was performed to correlate cfu/ml with Ct values for each primer set as follows. Cultures were grown to OD_600_ = ∼0.5 and serially diluted 10-fold. An aliquot from each dilution was spread on TSB agar plates and grown overnight to determine CFU/ml. 1 ml was used to extract genomic DNA using QIAGEN's QIAMP DNA mini kit and 2 µl used as template for qPCR. Standard curves were used to determine genome equivalents for Ct values obtained from aged dormant samples.

## Results

### *Resuscitation with spent medium allows an increase in bacterial growth from cultures of S. aureus SH1000 in a cold starvation model*

Over the course of 30 days the number of culturable cells in the cultures fell from between 10^6^–10^7^ cfu/ml to below 10 cfu/ml as measured by MPN tests in liquid media, whilst the total count as determined by microscopy remained around 10^6^–10^7^ cfu/ml.

**Figure 1:**
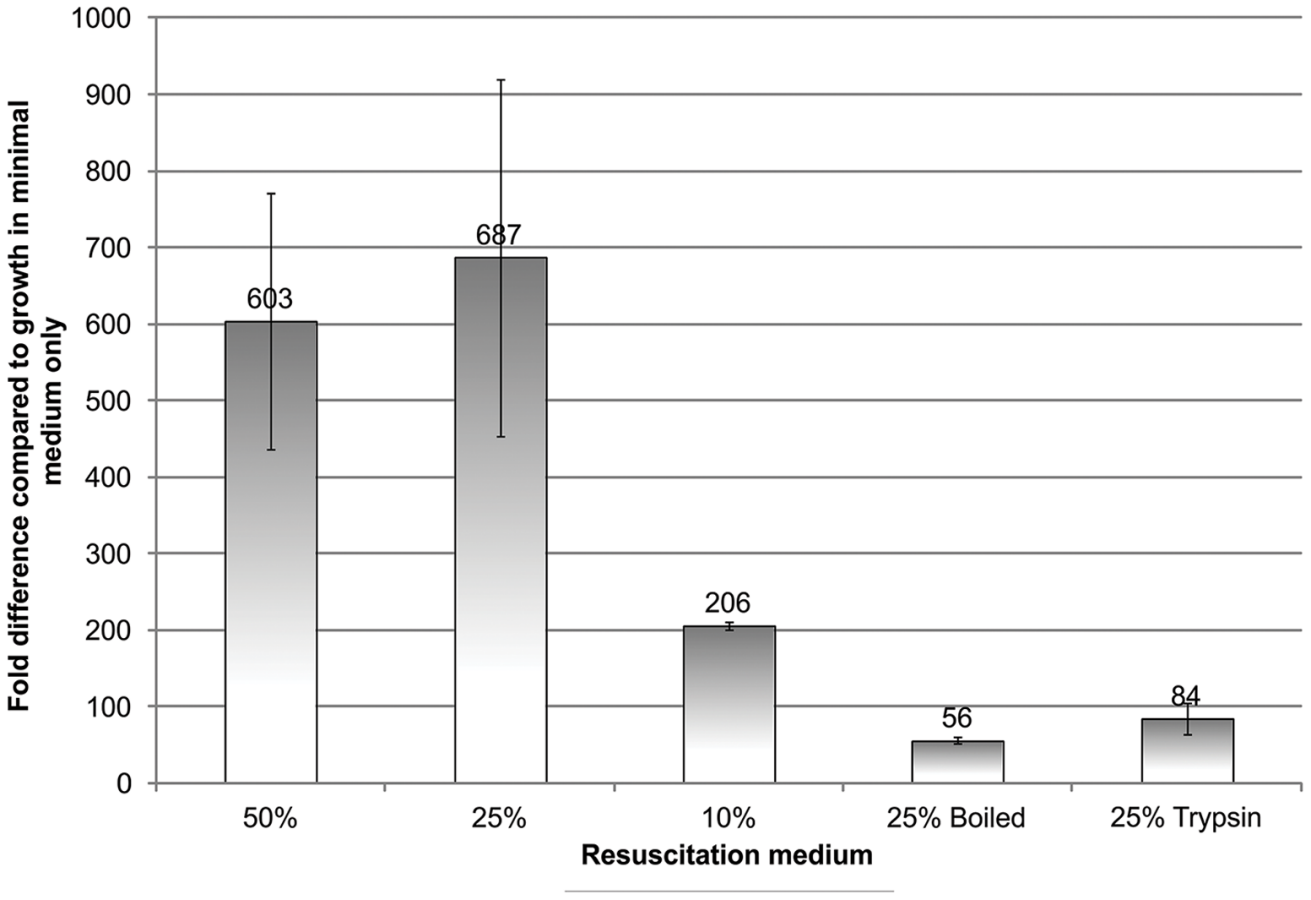
Resuscitation of dormant *S. aureus* SH1000 following addition of spent medium supernatant. Sterile filtered spent medium supernatant was added to fresh minimal medium inoculated with cells from 14 day old dormant cultures in MPN tests, and read at 24%, 25% and 10% concentrations of spent medium supernatant, and 25% boiled or trypsin-digested supernatant are shown. Aliquots of 100 µl of bacterial cultures from the dormant model were used to inoculate 900 µl fresh minimal medium supplemented with spent medium supernatant. The ratio of spent medium supernatant to fresh minimal medium was 100 µl: 800 µl for experiments using 10% spent medium supernatant, 250 µl: 650 µl for experiments using 25% spent medium supernatant and 500 µl: 400 µl for experiments using 50% spent medium supernatant. Trypsin-digested supernatant was prepared by digesting with 50 µg/ml trypsin from bovine pancreas for 30 minutes at 37°C; digestion was stopped by addition of 100 µg/ml type I-S trypsin inhibitor from soybean. Controls used 25% unboiled/undigested supernatant. Averages from three experiments and standard error bars shown in each case.

Resuscitation was observed when supplementing fresh defined media with 10–50% spent medium, with the peak effect using spent medium from cultures grown to an optical density of OD_600_ between 0.8–1.2. On average using 50 or 25% spent medium an over six-hundred-fold increase in bacterial growth at 24 hours was observed as measured by MPN method (range 200–900-fold), a statistically significant difference (Fig. 1) compared to the negative controls, demonstrating also that dormant cells, which could be cultured only after resuscitation, were present, and therefore that the number of viable bacteria was greater than the number culturable by standard methods. Whilst the difference between the effect with 50% and 25% supernatant was not significant, reducing the proportion of supernatant from 25% to 10% reduced the resuscitation effect, which disappeared completely when less than 10% spent medium supernatant was added. In the control in which spent medium supernatant and fresh minimal medium were incubated without inoculating with dormant cells, no growth was seen (data not shown).

**Figure 2:**
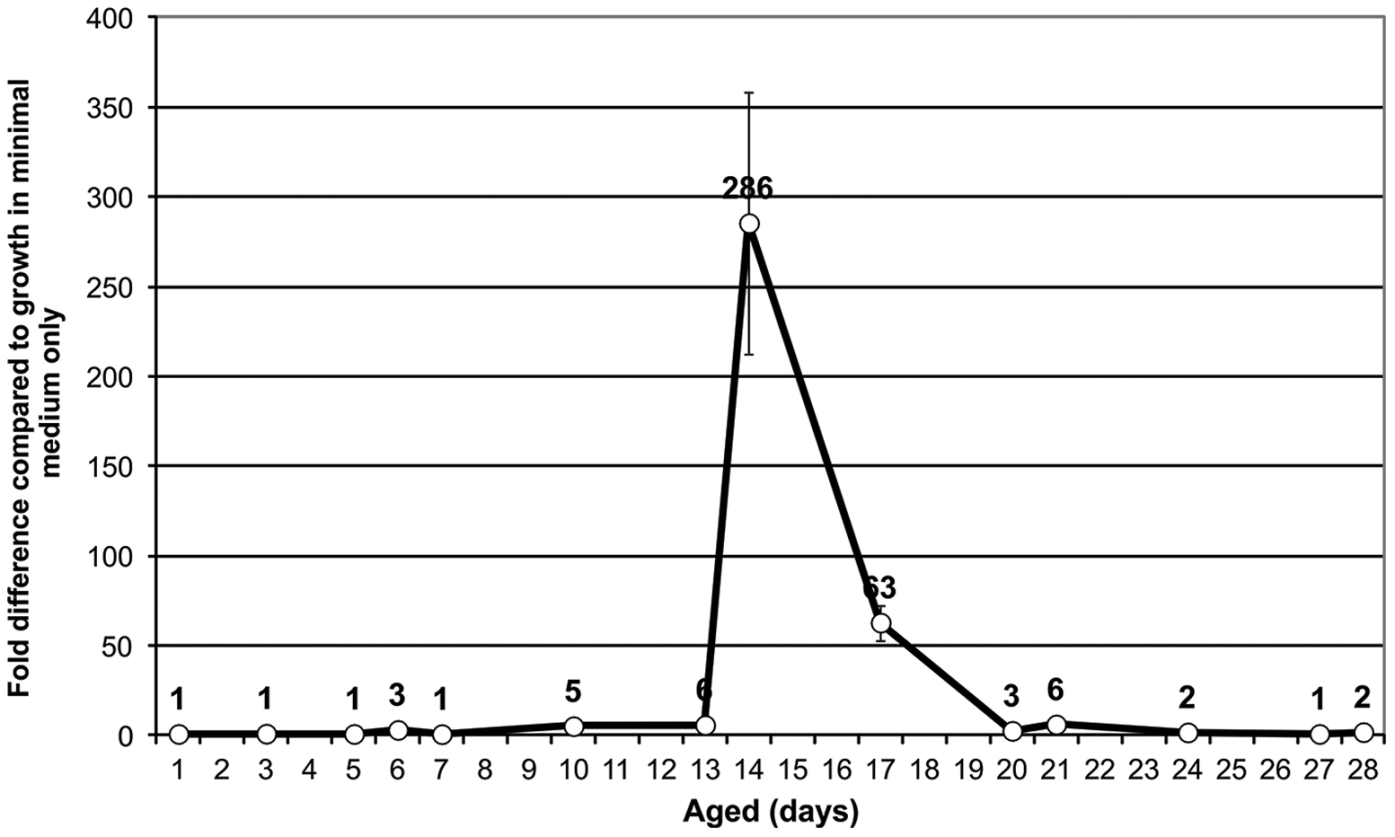
Effect of addition of 25% spent medium on dormant cultures of different ages. Spent medium was harvested from the SH1000 wild type *S. aureus* strain grown in minimal medium until late log phase. Cells were removed by centrifugation and then the supernatant was sterile-filtered with an Ø 0.22 µM filter unit. Aliquots of 100 µl of bacterial cultures from the dormancy model at different time points were used to inoculate 650 µl fresh minimal medium supplemented with 250 µl spent medium supernatant. Cultures were serially diluted in tenfold dilutions to perform 5-tube MPN tests, incubated at 37°C for 24 hours. Average of 3 independent experiments, with error bars.

**Figure 3:**
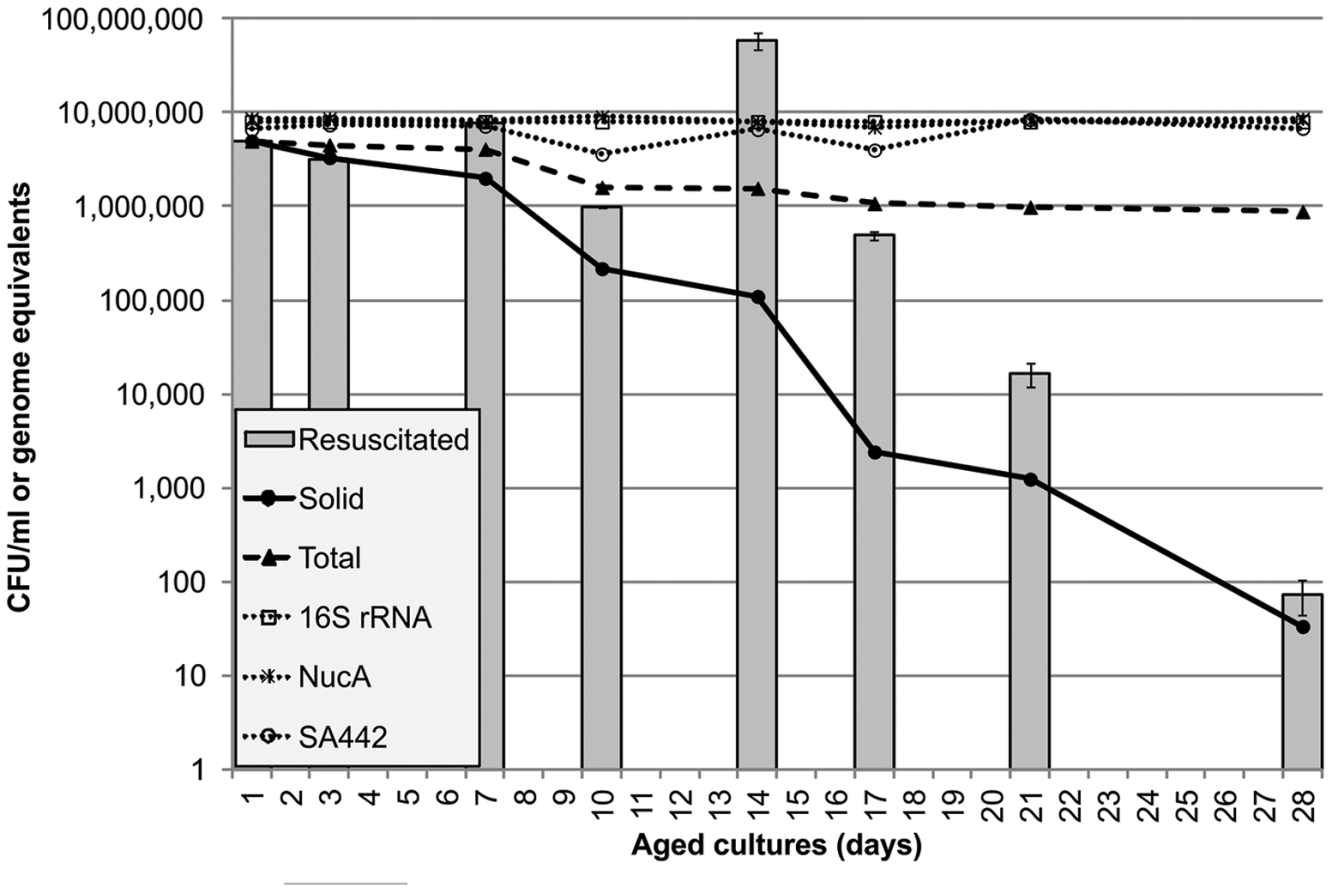
Bacterial counts in resuscitated cultures. Dotted lines: total count by microscopy (▴); genomic DNA content as determined by qPCR using 16S rRNA (□), NucA (*) and Sa442 (○) primer sets. *Solid lines*: total culturable counts, in liquid culture by MPN tests (▪) and on solid agar (•). *Bars* represent the estimated viable count in MPN tests after resuscitation –a figure obtained by multiplying the counts in MPN tests without resuscitation by the fold change in growth following resuscitation with 25% spent medium (averages from three experiments with standard error bars).

It was observed that only cultures of a particular age could be resuscitated. By taking aliquots and testing cultures throughout their incubation period it was demonstrated that cultures aged around 14 days were most responsive to resuscitation (Fig. 2). At 14 days, there were c.10^5^ viable cells; c.10^7^ genome equivalents were detected, meaning that c. 1% of the genome equivalents were viable. 99–99.9% of the cells could be resuscitated but were not culturable (Fig. 3). Continuing culture up to 70 days showed that cultures displayed no further resuscitation up to that time-point (data not shown).

### Resuscitation raises the culturable cell count of S. aureus SH1000 to near the total count during the period of maximum resuscitatability

By resuscitating the dormant cultures using spent medium, it was possible to increase the viable count to approach the total count (by microscopy) in cultures up to 14–17 days old. This is seen in figure 3, where the total observed count (by microscopy) is shown, together with the actual growth in liquid culture without resuscitation, as estimated by MPN tests, and the estimated number of cells which grew in the same tests with resuscitation - a figure obtained by multiplying the counts in MPN tests without resuscitation by the fold change in growth following resuscitation with 25% spent medium. These data imply that although many cells present were non-culturable by standard methods, they could still be resuscitated. After this, resuscitation was not able to allow all the cells to grow, suggesting that they may either be dead, or require resuscitation by an alternative procedure. The estimated number of cells given when performing quantitative real-time PCR with the NucA, 16S rRNA and Sa442 primer pairs supported the observed number of cells seen under the microscope (Fig. 3). Up to 28 days there appeared to be minimal decline in cell numbers, despite viable counts decreasing substantially. As well as supporting the microscopy results regarding total cell number these results also served as reassurance that the growth detected was of *S. aureus*, and not contaminating bacteria, since *nucA* and *Sa442* are *S. aureus* specific. Additionally, since RT-PCR using primers for FemA-SE, which is specific for *S. epidermidis*, was negative (not shown), and since the presence of similar numbers of cells was indicated using both the primers specific to *S. aureus* and those for the ubiquitous 16S rRNA gene, contamination with a second undetected species (a mixed culture) is unlikely.

### Resuscitation with spent medium reduces the lag phase of dormant cultures

The lag phase of dormant cultures was measured in the Bioscreen optical growth analyser. The figures used are from 3 separate runs each containing 5 replicates. There was a statistically significant difference in lag phase between those supplemented with spent medium and controls for dormant cultures aged 7 days (p = 0.0005), 10 days (p = 0.0139), and 14 days (p = 0.0010) which was lost with older dormant cultures aged 21 days (Table 1). Effects on lag phase were noted to appear a few days sooner than the effect on resuscitation of dormant cells.

**Table 1:** Results of resuscitation experiments using the Bioscreen system and *S. aureus* SH1000 dormant cultures.

Age of culture (days)	Mean change in lag phase using spent supernatant vs controls (hours)	Standard deviation	p-value
7	10.5	8.5	0.0005*
10	13.1	19.9	0.0139*
14	5.7	5.7	0.0010*
21	−0.5	5.0	0.1563

* indicates statistically significant result.

**Figure 4:**
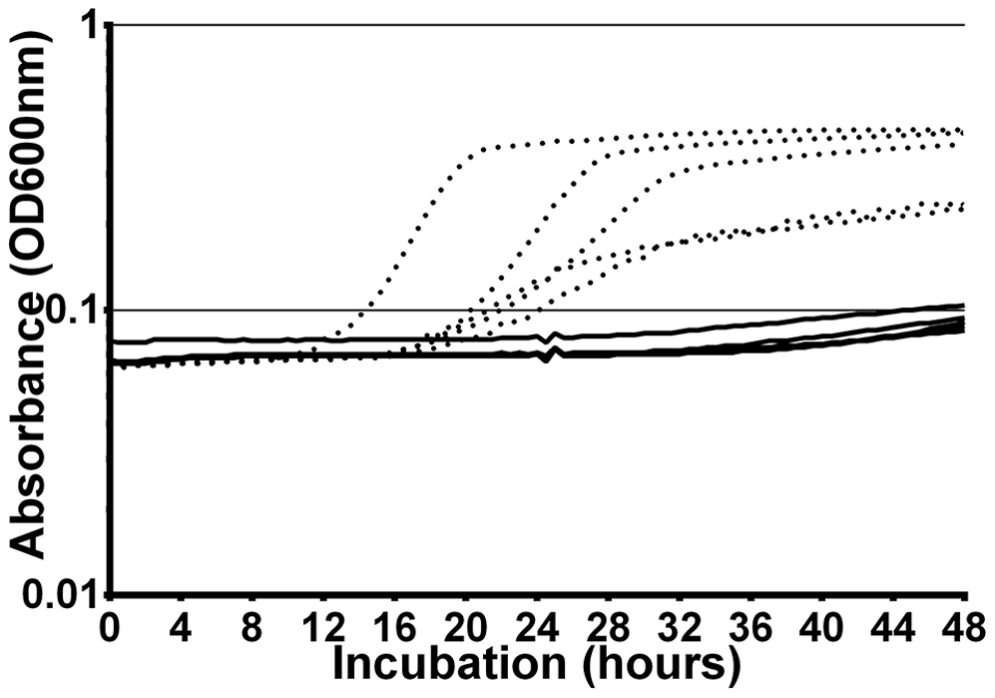
Differences in lag phase between cultures with and without the addition of spent medium supernatant. Measured by the Bioscreen instrument: example of one experiment. Dormant cells of SH1000, aged 7 days, with and without addition of 50% spent medium supernatant derived from SH1000 cultures: five replicate cultures. *Solid black lines*: increasing absorbance values of aged cultures inoculated in fresh minimal medium without supplementation. *Dashed lines*: increasing absorbance values of aged cultures inoculated in supplemented fresh minimal medium. Difference in lag phase as indicated by OD600 reaching >0.1 was measured.

Fig. 4 shows an example of a Bioscreen run using 7-day old SH1000 dormant cultures with and without supplementation with 50% spent medium.

### Boiling or trypsin digestion of supernatant results in loss of ability to resuscitate

Boiling the spent medium greatly reduced the resuscitation effect in the MPN tests although some activity remained (Fig. 1), and the same was true after digestion with trypsin. Averages and standard errors are shown in Fig. 1. The difference between the resuscitation levels seen following addition of 50% or 25% spent medium, and those with 25% boiled or trypsin-digested medium, was statistically significant (p = 0.008). Boiling of the supernatant also resulted in loss of activity of supernatant in the experiments measuring lag phase (data not shown).

### Greatest resuscitation activity is in the 10–30 kDa fraction

**Figure 5:**
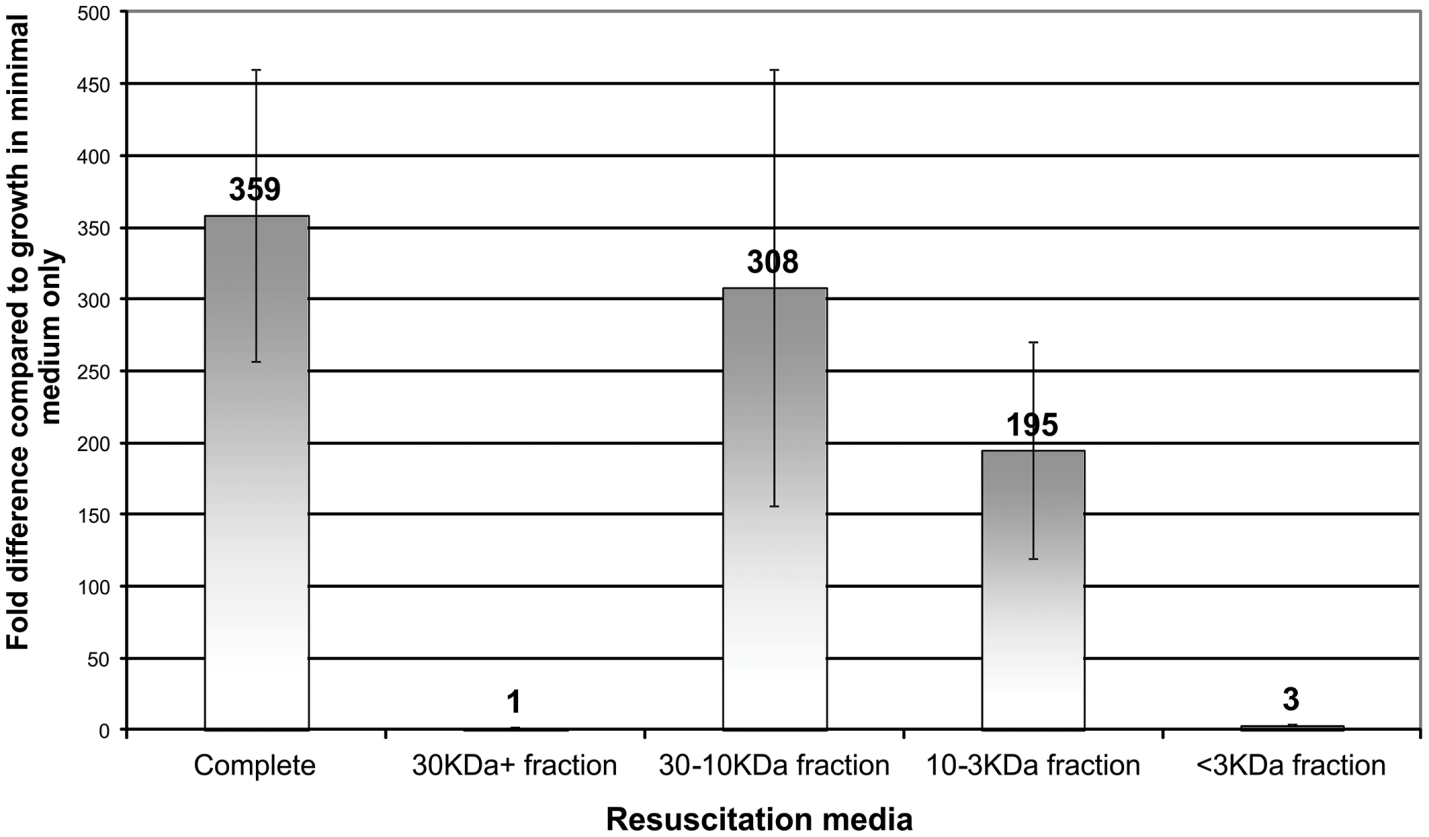
Resuscitation of dormant cultures following addition of 25% fractions of ultrafiltrated spent medium supernatant. 30+ fraction, 30–10 kDa fraction, 10–3 kDa fraction and <3 kDa fractions were tested, using MPN tests, on 14 day old dormant cultures inoculated into minimal medium. Averages from three experiments and standard error bars shown.

Ultrafiltration of the supernatant showed that the greatest resuscitation activity, measured by resuscitation in MPN tests, was in the 10–30 kDa fraction, with activity also seen in the 3–10 kDa fraction (Fig. 5). The resuscitation activity of the complete fraction, the 30–10 kDa fraction and the 10–3 kDa fraction was statistically significantly greater than that of the 30+ kDa fraction and the <3 kDa fraction, (p<0.05) (Figure 5).

### SEM appearance of cells from the dormant model

**Figure 6:**
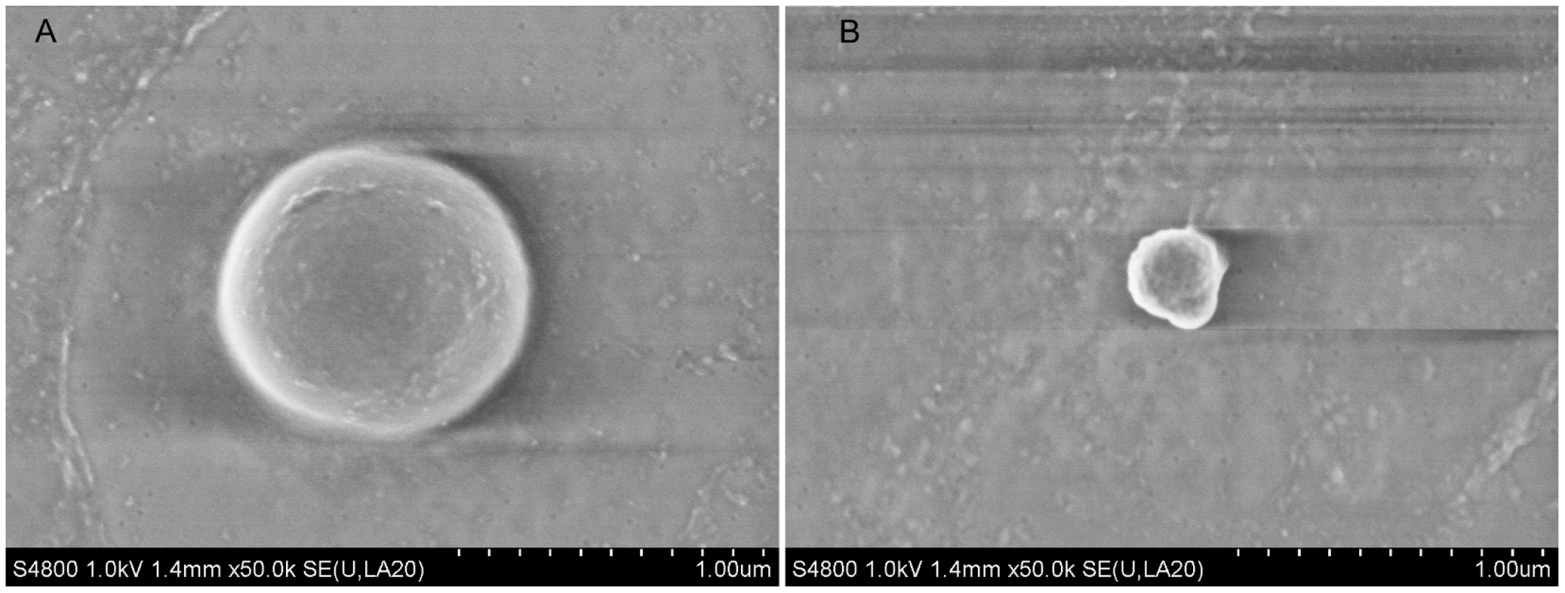
Dormant cells aged 7 days viewed under the SEM. The appearances of fresh and older cultures were compared. In fresh cultures almost all cells appeared as in (A): increasing amounts of smaller cells (B) were present in older cultures. Hitachi S4800 microscope used, ×50,000 magnification, uncoated at low voltage (1 kV).

Figure 6 shows the SEM appearance of cells from the dormant model examined after 7 days incubation. In fresh cultures, only the larger, typical coccoid staphylococcal cells (Fig. 6A) (diameter approximately 1 µm) were visible. With longer incubation, they became more difficult to find in the cultures, whilst smaller (diameter approximately 0.35–0.5 µm) apparently coccoid structures started to become visible (Fig. 6B). The older the dormant culture, the more of these small structures were visible and the greater their proportion relative to the larger staphylococcal cells. In fresh samples 100% of the cells observed were of the typical 1 µm diameter whilst after 7 days 80% of the cells observed were smaller cells, losing their smooth appearance and becoming irregular and pitted in appearance. After 14 days, the larger typical-looking cells were few and far between. Aged cells did not bunch together as much as fresh cells. Examination of tryptone soy broth inoculated with water rather than cells revealed no cells or other small coccoid structures similar to those described.

### Other *S. aureus* strains

Supernatant from all eight strains could resuscitate at least some other strains, and all eight strains could be resuscitated by certain supernatants (Table 2). Supernatant from SH1000 cultures was able to resuscitate dormant cultures of all the seven other tested strains, and supernatant from all the seven other strains could resuscitate dormant cultures of SH1000. Supernatants from all strains except MRSA252 and Col could resuscitate their own dormant cells. In general, supernatants from the clinically-derived strains had a less broad ability to resuscitate many other strains than those derived from 8325-4, SH1000 and SA113, which are closely related to one another, and the clinically-derived strains were less likely to be resuscitated by other supernatants, with supernatant from the Col strain showing the least activity. The full results are shown in Table 2. Experiments were performed three times with 5 replicates in each run.

**Table 2:** Results of resuscitation experiments with different strains of *S. aureus*.

Resuscitation medium									
	SH1000			8325-4			SA113		
	Mean change in lag phase (h)	SD	p-value	Mean change in lag phase (h)	SD	p-value	Mean change in lag phase (h)	SD	p-value
SH1000	20.3	8.9	0.0001	19.6	13.6	0.0032	18.7	14.5	0.0016
8325-4	18.0	14.0	0.0032	18.4	12.0	0.0005	15.5	9.2	0.0032
SA113	3.4	4.9	0.0156	12.9	11.0	0.0002	4.3	7.3	0.0625
RN4220	11.7	11.5	0.0010	12.7	10.7	0.0010	1.6	3.4	0.1250
MW2	20.9	10.8	0.0005	21.5	8.1	0.0001	8.1	11.3	0.0098
MRSA252	4.9	6.4	0.0078	2.6	5.4	0.0938	5.8	8.9	0.0313
USA300	11.1	9.5	0.0095	14.1	12.6	0.0005	15.0	7.5	0.0001
Colindale	9.0	7.0	0.0001	6.2	6.5	0.0020	1.6	3.6	0.0625

Resuscitation medium									
	RN4220			MW2			MRSA252		
	Mean change in lag phase (h)	SD	p-value	Mean change in lag phase (h)	SD	p-value	Mean change in lag phase (h)	SD	p-value
SH1000	8.1	11.4	0.0439	12.5	10.1	0.0001	9.8	9.4	0.0095
8325-4	10.2	8.4	0.0010	8.3	10.4	0.0010	9.5	8.9	0.0001
SA113	0.7	2.2	0.2500	6.3	10.6	0.0938	2.2	7.2	0.3125
RN4220	3.4	3.9	0.0098	2.7	3.4	0.0313	8.1	12.0	0.0806
MW2	10.8	8.4	0.0010	3.5	5.6	0.0313	13.0	12.4	0.0010
MRSA252	5.9	9.9	0.0313	2.3	4.0	0.0625	−1.4	12.2	0.2500
USA300	2.4	4.0	0.1094	8.3	10.3	0.0010	4.8	9.8	0.0032
Colindale	−1.0	12.9	0.5000	−0.2	0.8	0.2500	2.2	10.5	0.2344

. Resuscitation medium									
	USA300			Colindale					
	Mean change in lag phase (h)	SD	p-value	Mean change in lag phase (h)	SD	p-value			
SH1000	7.5	12.5	0.0039	6.8	8.6	0.0439			
8325-4	4.3	8.9	0.0938	9.0	10.4	0.0039			
SA113	0.2	0.8	0.5000	−2.0	8.6	0.5000			
RN4220	4.5	6.8	0.0313	1.5	2.2	0.0313			
MW2	12.6	10.9	0.0020	7.3	6.6	0.0010			
MRSA252	7.8	13.8	0.0156	0.3	1.0	0.5000			
USA300	5.1	6.8	0.0098	2.3	3.7	0.1094			
Colindale	0.0	0.0	1.0000	0.3	1.2	0.5000			

SD = standard deviation.

Mean change in lag phase is the difference in time taken for the supplemented vs unsupplemented cultures to reach the OD600 = 0.1 threshold in the Bioscreen growth analyser.

## Discussion

We have established and characterized a staphylococcal dormancy model and demonstrated for the first time that dormant cells of *S. aureus* can be resuscitated by spent culture medium. After reducing the viable count in staphylococcal cultures using a cooled nutrient-starvation model, a proportion of the cells can be resuscitated by adding sterile filtered culture supernatant from late-log phase cultures, with very similar findings to those with Rpfs from the actinobacteria. As with actinobacteria and Rpfs, the same spent medium can also reduce the lag phase when dormant cultures are sub-cultured into minimal medium. The greatest activity when testing for reduced lag phase was seen in dormant cultures aged 7–14 days; when using MPN, the greatest effect was seen at around 14 days. Results displayed some variability in the extent of resuscitation under apparently identical conditions. To control for this variability multiple experiments were performed and mean values stated in the respective experiments. This variability accounts for the difference in fold resuscitation seen in figures 1, 2 and 5. For each experiment, all results were subjected to statistical analysis to confirm statistically significant differences with the controls.

Both resuscitation and shortening of lag phase are greatly reduced by boiling the supernatant, or by trypsin digestion, and is greatest in the 3–30 kDa fractions of the supernatant. This suggests that the active component(s) in the supernatant are likely to be protein/peptide factors secreted by actively growing cultures. By resuscitating the dormant cultures, it was possible to increase the viable count to approach the total count (by microscopy), implying that although the cells present were non-culturable by standard methods, they could still be resuscitated. After this, resuscitation was not able to allow all the cells to grow, suggesting that they might be either dead, or require resuscitation by an alternative procedure.

These findings display many similarities to those with Rpfs in the actinobacteria. The Rpf of *Micrococcus luteus* has a molecular mass of 16–17 kDa and those of *M. tuberculosis* range from 15–37 kDa. In *M. tuberculosis*, which has five Rpf homologues, some Rpfs have a greater potency in shortening lag phase and others in their effect on cell culturability^11^, whilst the Rpf of *M. luteus* has both effects^8^. Bioinformatic analysis of the *rpf*-gene of *M. luteus* indicates a LysM module at the C-terminus, which probably promotes its association with the cell envelope peptidoglycan [30]. The active domain in Rpfs is distantly related to c-lysozyme [31] and they have muralytic activity [32], [33]. RpfB of *M. tuberculosis* interacts and synergizes with Rpf-interacting protein A (RipA), an endopeptidase, to hydrolyze peptidoglycan and facilitate growth [34]. Combined action with other peptodoglycan hydrolases might produce muropeptides that may exert biological effects through bacterial or host signaling [35]. Depletion of RipA resulted in increased susceptibility to the cell wall-targeting beta-lactams [36], and the peptidoglycan-synthesizing enzyme, penicillin binding protein 1 (PBP1), has been shown to interact with RipA, inhibiting the synergistic hydrolysis of peptidoglycan by the RipA-RpfB complex in vitro [37]. A *Mycobacterium tuberculosis* mutant lacking Rpfs had enhanced sensitivity to beta-lactam antibiotics [38]. All these findings support the suggestion that the dormant organisms may undergo cell-wall changes, possibly with inert peptidoglycan which requires scission by Rpf or its associated proteins in order to resume growth and replication. A similar mechanism is possible in staphylococci, though it is by no means the only likely scenario. It has been noted that germination of *Bacillus subtilis* spores can be induced by a muropeptide, that may bind to a well-conserved Ser/Thr membrane kinase capable of binding peptidoglycan [39], and that *B. subtilis* expresses and secretes a muralytic enzyme, YocH, in response to cell wall-derived muropeptides derived from growing cells [40].

Although Rpfs themselves are not found in firmicutes, it is postulated that proteins with a related function may exist in this group and recent data from studies of *Listeria monocytogenes* supports this [15]. Rpf proteins have been classified into several subfamilies by *in silico* analysis of the accessory domains [12]. The RpfB subfamily has very similar domain structures and genomic contexts to a group of proteins of unknown function in firmicutes, including the staphylococci. In these proteins, the Rpf domain is replaced by another completely different domain designated Sps (stationary phase survival). Forty-six Sps domains have been identified, and they are found to be associated with other domains known to be present in muralytic enzymes (eg SH3b and LysM). The sequence similarity between the C-terminal region of the Sps domain and that of the Gram-negative lytic transglycosylase MltA also supports the association. Therefore, it has been postulated that proteins containing the Sps domain may perform a similar function to Rpfs [12]. In staphylococci, two such proteins have been been identified [12]: SceD and SceA in *S. carnosus*, which have orthologues in *S. aureus* and *S. epidermidis.* What is known about these proteins? The SceA orthologue in *S. aureus* has been designated IsaA (immunodominant staphylococcal antigen), identified as a dominant antigen in human MRSA sepsis [41]. It is reported to include a putatative soluble lytic transglycosylase domain in its C-terminal region [42]. IsaA is found both in the culture supernatant and in the cell-wall fraction of *S. aureus* cultures, specifically, located on the septal region of the cell surface [41], suggesting that it may be involved in cell separation. Like IsaA, SceD also demonstrates cell wall hydrolytic activity, and cleaves peptidoglycan [43]. Regulatory studies have shown *sceD* and *isaA* to be mutually compensatory [43], with inactivation of *isaA* leading to increased transcription of *sceD -* they may have overlapping, (but distinct) roles [15]. IsaA is one of the genes controlled by the YycG/YycF two-component regulatory system in *S. aureus* [44]; *sceD* is also included in the YycFG regulon [15]. YycF binds specifically to the promoter regions of IsaA and LytM, consistent with the proposed role of the system in controlling cell wall metabolism and virulence [44]. In a comparative proteome analysis of *S. aureus* biofilm and planktonic cells, and correlation with transcriptome profiling, biofilm cells were found to be expressing much lower amounts of IsaA than were planktonic cells [45]. Much of this evidence again mirrors findings with Rpf in *M. luteus* and mycobacteria. It points towards a possible role for IsaA in the control of exit from stationary phase. Although the exact role of *sceD* is unknown, there is an abundance of *sceD* transcripts in both hVISA and VISA strains of *S. aureus* , which correlates with increased cell wall turnover and altered cell-wall structure [46], [47]. In preliminary experiments not presented here, we found that supernatant from *S. aureus* SH1000 was able to resuscitate dormant cells of the *ΔisaA* and *ΔsceD* mutants of *S. aureus* SH1000 [43]: supernatant from both mutants showed a loss of ability to resuscitate cultures of the other, although they could both resuscitate dormant cultures of SH1000. Further work is required to investigate the possible role of IsaA and SceD in resuscitation, by purifying the proteins and testing their growth induction properties directly.

SEM of the dormant cultures reveals the presence of small (0.35–0.5 um) coccoid structures, resembling bacteria. Cells entering the viable but non cultivable state are recognized to exhibit dwarfing [48], so we postulate that the small coccoid structures may represent ‘dormant’ staphylococcal cells, a situation analagous to debated changes in dormant cells of *Mycobacterium tuberculosis*, which have been reported to take ovoid forms [49], and to undergo marked cell wall thickening [50] under challenging conditions. However to date we have no firm evidence that the small coccoid structures we have demonstrated are staphylococcal forms, despite their absence from control samples.

Although supernatant from all eight strains tested could resuscitate at least some other strains, and all eight strains could be resuscitated by certain supernatants, generally the clinically-derived strains were less active in resuscitating other strains and less likely to be resuscitated by other supernatants. It is tempting to speculate that this relative lack of both resuscitability and ability to resuscitate may be related to persistence in a clinical setting. In principle, if dormant cells could be returned to a higher state of metabolic activity, or ‘resuscitated’, they might become more susceptible to antibiotics: bacterial antibiotic susceptibility increases with increased growth rate [51], [52] and the action of aminoglycosides against bacterial persisters was shown to be potentiated by metabolic stimulation [53]. Ability to treat these and organisms in biofilms could lead to less treatment failures, less prolonged antibiotic use, less need for high-dose antibiotic regimens and ultimately less selection for genetic resistance. Further work is required, to determine whether these results are specific to this method of inducing dormancy or whether they also hold for methods which use antibiotic killing; and also to elucidate the active component(s) in the supernatant, and mechanism of action, and eventually to determine whether addition of resuscitating factors can increase antibiotic susceptibility as well as growth, potentially giving us an insight into how treatment of these difficult and chronic infections could be improved.

## Supporting Information

Table S1 **List of primers used.**
(DOCX)
