# Supplementary material for: Dormant Cells of Staphylococcus aureus Are Resuscitated by Spent Culture Supernatant
Source: PLoS One. 2014 Feb 11;9(2):e85998. doi: 10.1371/journal.pone.0085998 (PMC3921112; doi:10.1371/journal.pone.0085998)
Supplement: Table S1 — List of primers used. (DOCX) [file pone.0085998.s001.docx]

**Table S1**

| **Primer** | **Sequence** | **Source** |
| --- | --- | --- |
| Sa442_F | GTC GGG TAC ACG ATA TTC TTC ACG | Reischl *et al.* 2000 |
| Sa442_R | CTC TCG TAT GAC CAG CTT CGG TAC | Reischl *et al.* 2000 |
| NucA_F | GCG ATT GAT GGT GAT ACG GTT | Brakstad *et al.* 1992 |
| NucA_R | AGC CAA GCC TTG ACG AAC TAA AGC | Brakstad *et al.* 1992 |
| 16S rRNA_F | CAT TTC ACC GCT ACA CAT GG | Designed for this study |
| 16S rRNA_R | TTA TGG AGA GTT TGA TCC TGG C | Designed for this study |
